# Generating polyketide diversity in *Dictyostelium*: a Steely hybrid polyketide synthase produces alternate products at different developmental stages

**DOI:** 10.1098/rspb.2022.1176

**Published:** 2022-09-28

**Authors:** Tamao Saito, Tomoyuki Iijima, Kohei Koyama, Tomonori Shinagawa, Ayaka Yamanaka, Tsuyoshi Araki, Noriyuki Suzuki, Toyonobu Usuki, Robert R. Kay

**Affiliations:** ^1^ Faculty of Science and Technology, Sophia University, Tokyo 102-8554, Japan; ^2^ Graduate School of Science and Technology, Sophia University, Tokyo 102-8554, Japan; ^3^ MRC Laboratory of Molecular Biology, Cambridge CB2 0QH, UK

**Keywords:** *Dictyostelium discoideum*, polyketide synthase, dibenzofuran, differentiation-inducing factor (DIF), antibacterial compound

## Abstract

The soil is a rich ecosystem where many ecological interactions are mediated by small molecules, and in which amoebae are low-level predators and also prey. The social amoeba *Dictyostelium discoideum* has a high genomic potential for producing polyketides to mediate its ecological interactions, including the unique ‘Steely’ enzymes, consisting of a fusion between a fatty acid synthase and a chalcone synthase. We report here that *D. discoideum* further increases its polyketide potential by using the StlB Steely enzyme, and a downstream chlorinating enzyme, to make both a chlorinated signal molecule, DIF-1, during its multi-cellular development, and a set of abundant polyketides in terminally differentiated stalk cells. We identify one of these as a chlorinated dibenzofuran with potent anti-bacterial activity. To do this, StlB switches expression from prespore to stalk cells in late development and is cleaved to release the chalcone synthase domain. Expression of this domain alone in StlB null cells allows synthesis of the stalk-associated, chlorinated polyketides. Thus, by altered expression and processing of StlB, cells make first a signal molecule, and then abundant secondary metabolites, which we speculate help to protect the mature spores from bacterial infection.

## 1. Introduction

Soil supports a complex ecosystem fuelled by plant organic material and containing abundant bacteria, amoebae, fungi and invertebrates. The amoebae, which include slime moulds such as *Dictyostelium discoideum*, are low-level predators feeding primarily on bacteria, and themselves can fall prey to other amoebae, fungi and invertebrates, such as nematodes [1,2]. Many of the ecological interactions in soil are driven by the production of bioactive small molecules including chemoattractants and repellents, anti-feedants and antibiotics. Soil organisms, such as *Streptomycete* bacteria are therefore a rich source of drugs and antibiotics. Understanding these soil eco-chemical interactions is also important for understanding the soil ecosystem as a whole and maintaining its health [3].

The cellular slime mould, *D. discoideum,* lives in the soil and has a unique two-staged life cycle. During growth, the cells consume bacteria and yeasts and remain as unicellular amoeba; but when their food is depleted, they chemotax together to form a multicellular aggregate. Within this aggregate, cells differentiate into prestalk and prespore cells in a heterogeneous mixture and then sort out to form coherent prestalk and prespore tissues, as it transforms into a migrating slug. After the slug stage, cells complete differentiation, producing a fruiting body consisting of a stalk of vacuolated stalk cells, supporting a mass of dormant spores [4,5].

Sequencing the genome of *D. discoideum*, and subsequently other slime mould genomes, revealed a great abundance of polyketide synthases (PKSs), most of which are specialized to each species [6–8]. PKS proteins are flexible biosynthetic machines that condense acylated precursors—principally acetyl and malonyl-CoA—into polymers that can be reduced, cyclized and modified in other ways to give a great range of natural products [9,10]. The dictyostelid genomes, therefore, suggest that these amoebae are a rich source of polyketides, though to date only a few have been identified and their function and ecological roles remain uncertain.

The first polyketide to be identified from *D. discoideum*—DIF-1—is an acylated alkylphenone that is doubly chlorinated [11]. Rather than having an ecological function, it serves as a signal during the multi-cellular development of this organism: it induces stalk cell differentiation in monolayer assays and is required to form specialist cells supporting the fruiting body [12,13]. Elucidation of the biosynthetic pathway [15] showed that the polyketide precursor of DIF-1 (THPH: 2,4,6-trihydroxyphenyl-1-hexan-1-one) is made by a novel ‘steely’ PKS (StlB), which consists of a fusion of a type-I PKS/fatty acid synthase with a type-III PKS/chalcone synthase [14]. In this fusion, the type-I PKS is proposed to make a hexanoyl precursor, which it transfers over to the chalcone synthase domain for elongation and cyclization to give THPH.

StlB, and a second steely enzyme, StlA, that also makes a developmental signal molecule [16,17], are conserved across slime mould species [18], but have not been reported from other organisms. Steely enzymes have the potential to streamline polyketide synthesis and so are of biotechnological interest: indeed, StlB has recently been engineered to make cannabinoid precursors in *D. discoideum* [19].

To complete DIF-1 biosynthesis, the THPH polyketide is chlorinated by a flavin-dependent halogenase (ChlA) and O-methylated by a methyl transferase (DmtA) [20,21]. StlB and ChlA, but not DmtA, are adjacent in the genome and share a common promoter region.

Organic halogen compounds are widespread in nature, and most are thought to have an ecological function; they often have some kind of biological activity including antibacterial and anti-cancer activity [22]. Apart from DIF-1, other chlorinated compounds produced by cellular slime moulds include a polychlorinated dibenzofuran, AB0022A from *Dictyostelium purpureum*, which has antibacterial activity against Gram-negative bacteria; and two chlorinated dibenzofurans Pf-1 and Pf-2 from *Polysphondylium filamentosum* [23,24].

DIF-1 synthesis and metabolism were previously investigated by labelling cells metabolically with radioactive ^36^Cl^−^ [25]. These experiments showed, unexpectedly, that *D. discoideum* and other slime moulds also produce a set of abundant chlorinated compounds late during their development, which appear to be independent of DIF-1 and its metabolites. Their chemical identity, function and biosynthesis were unknown.

Here we return to these compounds—called CCDs in the original work [25]—and identify one of them as a chlorinated dibenzofuran, which we show has antibiotic activity. This compound accumulates in stalk cells late in development and our genetic evidence suggests that it is also made by the StlB and ChlA enzymes. Further, its production correlates with the appearance of a shortened form of StlB corresponding to the chalcone synthase domain. We, therefore, propose that StlB makes distinct polyketide compounds depending on the developmental stage and that this switch results from a change in expression from prespore to stalk cells and is assisted by processing of the protein to release the chalcone synthase domain.

## 2. Material and methods

### (a) *Dictyostelium* cells and transformation

*Dictyostelium discoideum* strain Ax2(Kay) [26] was used as parental stain and grown in HL5 axenic medium (Formedium) at 22°C. Transformants were cultured in HL5 containing blasticidin or G418 (10 µg ml^−1^) at 22°C. For development, axenically grown cells were washed and plated at 22°C on KK2 buffered (20 mM K_1_K_2_PO_4_, pH 6.2 with 2 mM MgSO_4_ added after autoclaving) agar plates at a density of 1–2 × 10^6^ cells cm^−2^. *Dictyostelium discoideum* strain V12M2 was used as the source of chlorinated metabolites for structural analysis, since it produces about 5 times more than A×2 cells (electronic supplementary material, figure S1). It was cultured on SM agar (Formedium) plates with *Klebsiella aerogenes* and the fruiting bodies collected.

The strategy used to knock-in a Tandem Affinity Purification (TAP) [27] tag at the 3′ end of the *stlB* coding region is summarized in electronic supplementary material, figure S2. About 1.6 kbp of the 3′ region of the *stlB* coding sequence with 0.45 kbp non-coding 3′ region was amplified by PCR with primers TS1-Apa and TS3-HindIII and a TAP-tagged coding region amplified with primers TS-TAP and TS anti-TAP. Primer TS-TAP contains an XhoI site and 4 x glycine coding sequence as a linker between the TAP tag and *stlB* C-terminal region. The termination codon of *stlB* was removed and the TAP Tag inserted in the XhoI site that was created in the SwaI site at the 3′ end of *stlB* gene. Knock-in of the TAP-tag was confirmed by PCR and continued production of chlorinated compounds by ^36^Cl^−^ labelling (electronic supplementary material, figure S3).

The strategy used to express the TAP-tagged chalcone synthase domain of StlB driven by the stalk-specific ST promoter is summarized in electronic supplementary material, figure S4. The tagged chalcone synthase domain of *stlB* was amplified by StlB-pks3-fw-BamHI and StlB-pks3-rv-XhoI primers using genomic DNA from the StlB-TAP-tag knock-in strain as template. The PCR product was digested with BamHI and Xho I and triple ligated in the BglII and XhoI site of the ST-gfp plasmid. The construct was confirmed by DNA sequencing and expression of the protein by western blotting.

To analyze the spatial expression of *stlB* and *chlA,* their promoter regions were amplified by PCR and ligated into pDXA3CΔ-EGFP after removal of the Actin 5 promoter. Spatial expression of GFP was examined by fluorescence microscopy (Axiovert135, Zeiss). The PCR product from the *stlB* promoter region is 2831 bp long, including 30 bp of the coding region of *chlA*. The *chlA* promoter region is 1781 bp, including 77 bp of coding region. Primers used in this study are summarized in table 1.

**Table 1.** Summary of primers used in this study.

StlB promoter
StlB-SalI: AAATGTCGACATCATAATGATTAATAATATTTGTATCCAT
StlB-XhoI: AAATCTCGAGTAAATCGTTTATACTTTTGTTGTTGTTCAT
ChlA promoter
ChlA-PstI: TTTTCTGCAGCGGTATTTCACTAAAAAGAGGC
ChlA-BamHI: AAAAGGATCCTGTCTAGTTGCTGATAATCCTGC
SteelyB-TAP fusion construct
SteelyB Type III PKS
TS1-Apa: AATGGGCCCGAATTAAAAGATAAAGATTT**G**AATAATACAGTATCA
TS3-Hind: TTAAAGCTTATTTTTTGTATAAGGATGTTCAGTGATTC
G: site directed mutagenesis to remove SwaI site without changing amino acid sequence; this mutation was used for the first screening of the mutants
SteelyB downstream
TS5-Bam: TTAGGATCCGCCTTTTCACCTGGTGCTTCAATTGAAGCA
TS19-Not: ATTAGCGGCCGCAACCAGTTTCAGTATCCATTCCATTAACAG
TAP-tag
TSTAP: AAATATCTCGAGGGTGGTGGTGGTGAAAAGAGATGGAAAAAG
AATTTCATAGCCG
antiTAP: AAATATTCTGAGTTAGGTTGACTTCCCCGCGGAATTCGCG
SteelyB chalcone synthase domain and TAP fusion protein, with ST promoter
StlB-pks3-fw-BamHI: GGATCCGAATTAACATCACCACCACCAAGT
StlB-pks3-rv-XhoI: CTCGAGGAGGGAGGAATCGAAGAAATAATC

### (b) Chlorine labelling, TLC and autoradiography

These were carried out as described previously [25]. Briefly, cells were developed on agarose plates (1.8% electrophoresis grade agarose) containing 10% DIFlab (100% is 12 mM KH_2_PO_4_, 8 mM Na_2_HPO_4_, 1 mM MgSO_4_, pH 6.7) with 0.1 µCi ml^−1^ ^36^Cl^−^. After appropriate times, cells were harvested, and organic components extracted by the Bligh and Dyer method [28]. Extracts were analysed on activated TLC plates (Whatman LK6D silica) developed with 60/40/2 ethyl acetate/hexane/acetic acid and visualized using a phosphoimager.

### (c) Western blotting

*Dictyostelium* cells were lysed directly in SDS sample buffer and proteins separated by electrophoresis on 5–20% gradient polyacrylamide gels (e-pagel, ATTO). Since the TAP-tag contains Protein A, Peroxidase Anti-Peroxidase (PAP) soluble complex from rabbit (Sigma-Aldrich 1 : 3000) was used to detect the TAP-tagged protein.

### (d) Purification of chlorinated compounds

Fruiting bodies of *D. discoideum* strain V12M2 were collected with a spatula and residual bacteria and spores removed by washing with SM buffer in a 250 µm mesh sieve. Mature stalks were extracted with ethanol at room temperature, the extract concentrated and then partitioned between ethyl acetate and water. The ethyl acetate extract was separated by preparative SiO_2_ column chromatography with a 4-step gradient solvent system: hexane-ethyl acetate (1 : 1), then hexane-ethyl acetate (1 : 3), pure ethyl acetate, pure acetone and finally pure methanol. The chlorinated compounds eluted mainly in the pure acetone fraction. Samples were further purified and analysed by reverse-phase HPLC (TSK gel ODS-120T) eluting at 1 ml min^−1^ with a gradient of 50–80% acetonitrile in 60 min. Column and detection temperature was maintained at 40°C. Purification was monitored by TLC, with compounds visualized by UV and phosphomolybdic acid. Compounds produced by Ax2 cells, but absent from *stlB*- mutant cells were purified initially and later characterized by mass-spectroscopy.

Detailed analysis of the purified chlorinated dibenzofuran-1 (CDF-1) was performed by MS (JEOL JMS-700) and NMR (JEOL JNM-ECA 500) and is summarized in electronic supplementary material, figures S5, S6 and S7. ^1^H-NMR and ^13^C-NMR spectra of CDF-1 are summarized in electronic supplementary material, table S1. X-ray crystallographic analysis is summarized in electronic supplementary material, table S2.

### (e) Polyketide feeding experiment

*StlB* null cells were harvested in late log-phase, washed with KK2 buffer and spread on 10% DIFlab agar containing 100 nM DIF-1 and 8 µM THPH or Cl-THPH and allowed to form fruiting bodies over three days. When fruiting body formation was complete, 5 ml of 8 µM THPH or Cl-THPH in DIFlab (10%) was spread on the 625 cm^2^ agar plate. The next day, fruiting bodies were collected, extracted with ethanol, partitioned between water and ethyl acetate and the ethyl acetate fraction taken. This was purified by SiO_2_ column chromatography, followed by HPLC. Reverse-phase HPLC elution was carried out at 1 ml min^−1^ with a gradient of 50–80% acetonitrile in 60 min as described above. About 1.5 × 10^10^ cells were used for each experiment.

### (f) Antibacterial activity

*Escherichia coli* B/r*, Bacillus subtilis* and *K. aerogenes* were used and the preparation is summarized in electronic supplementary material, figure S8.

Antibacterial activity was measured by Mueller-Hinton broth dilution. Serial two-fold dilution of Ampicillin (Positive control) and CDF-1 were prepared in 96 well plates. The highest Ampicillin sodium concentration was 128 µg mL^−1^ (that is 120 µg ml^−1^ concentration of Ampicillin). The highest concentration of CDF-1 was 100 µg ml^−1^. The diluted samples with bacteria were incubated in 96 well plate at 37°C for 16 h. The growth of the bacteria in each well was confirmed by microscopy. Three independent experiments were carried out and the highest dilution for growth inhibition was assigned as the minimal inhibitory concentration.

## 3. Results

### (a) Biosynthesis of all chlorinated compounds produced in *D. discoideum* development depends on the StlB PKS and ChlA chlorinating enzyme

Following earlier work, we used metabolic labelling with ^36^Cl^−^ to detect chlorinated compounds made during *D. discoideum* development [25]. Starving cells were plated at high density on agarose containing ^36^Cl^−^ and chlorinated compounds extracted from them with organic solvents at various times thereafter and examined by TLC and autoradiography.

**Figure 1.**
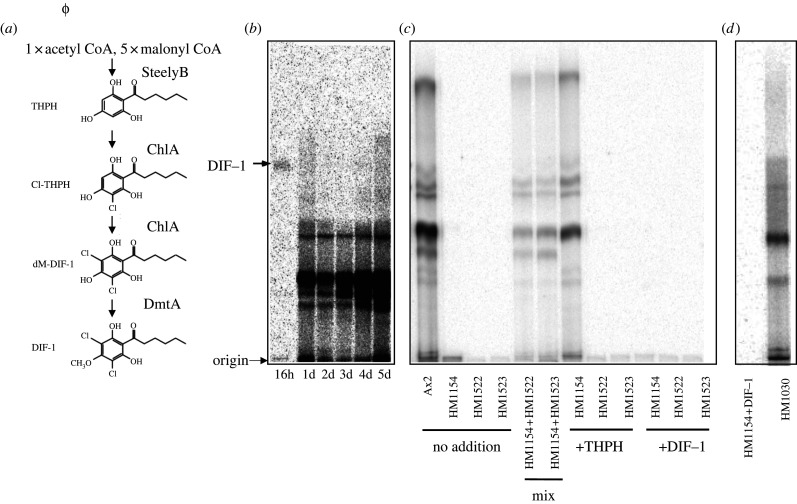
The polyketide synthase and chlorinating enzyme that produce DIF-1 also produce the abundant chlorinated compounds of fruiting bodies. (*a*) Biosynthetic pathway for DIF-1. DIF-1 is made from the polyketide THPH, which is produced by the SteelyB hybrid PKS, and then chlorinated and methylated by ChlA and DmtA. (*b*,*c*,*d*) Metabolic labelling experiments in which cells are developed on agarose containing ^36^Cl^−^ and extracts analysed by TLC and autoradiography. (*b*) Time course of chlorinated compound production during development. At 16 h, when cells have formed fingers and slugs, only DIF-1 is detectable. By 24 h fruiting bodies have formed and remain for the rest of the time course. (*c*) Genetic evidence that StlB and ChlA are required to produce the chlorinated compounds of fruiting bodies. ‘No addition’ lanes show that parental Ax2 cells produce abundant chlorinated compounds, while mutants lacking StlB (HM1154) or ChlA (HM1522 and HM1523) do not. ‘Mix’ lanes show that the mutants can synergize to restore production. ‘+THPH’ lanes show that production is rescued by supplying the polyketide THPH to *stlB^−^* but not to *chlA*^−^ cells. ‘+DIF-1’ lanes show that when proper development of the mutants is rescued by adding 100 nM DIF-1 to the agarose [13,14], production of the chlorinated compounds is not rescued. (*d*) The mutant lacking the DmtA methylase (HM1030) catalyzing the final step in DIF biosynthesis can still produce the late chlorinated compounds.

Development takes about 24 h, during which time the amoebae first aggregate together, then form a multicellular slug and finally a stalked fruiting body with spores on top. Figure 1*b* shows the time-course of chlorinated compound production: at 16 h, the slug stage, only DIF-1 is detectable—its metabolites are also made but are mainly released and below the detection threshold in cell extracts. By 24 h, little if any DIF-1 is detectable and the scene is dominated by a series of more polar compounds, which continue to increase for several days. These late compounds are largely confined to stalk cells, or released into the medium, and present at much lower levels in spore cells [25].

We investigated the biosynthesis of the late chlorinated compounds using null mutants of the three enzymes of DIF-1 biosynthesis and metabolic labelling with ^36^Cl^−^ (figure 1*c,d*). Deletion of the DmtA methyl transferase (strain HM1030) still permitted production of the late chlorinated compounds, indicating that it is not essential. A role in decorating these compounds cannot be totally excluded, even though *dmtA* is expressed in the spore region of the fruiting body [29], rather than the stalk where *stlB* and *chlA* are expressed. By contrast, no chlorinated compounds were detected in the absence of ChlA (strains HM1522 and HM1523), showing that it is the only relevant chlorinating enzyme.

Unexpectedly, deletion of the StlB polyketide synthase (HM1154) also ablated the production of all chlorinated compounds. We excluded two possible trivial explanations for this result. First, it could be that lack of DIF-1 in *stlB*- mutants, and consequent abnormal development of the fruiting bodies, prevents production of the late chlorinated compounds. To test this, we developed mutant cells on agar containing DIF-1, which restores normal development. However, production of the chlorinated compounds was not restored (figure 1*c*), eliminating this objection.

Second, the genetic manipulations required to create *stlB-* mutants may also have damaged the adjacent *chlA* gene, which shares a common promoter. To test this possibility, we performed a mixing experiment. *StlB*- and *chlA*- mutant cells were mixed (HM1154 and HM1522; HM1154 and HM1523) and allowed to develop together on agar containing ^36^Cl-. The results show clearly that production of the chlorinated compounds is restored, implying that the polyketide precursor produced by *chlA*- cells can cross into *stlB-* cells for chlorination and that the genetic manipulations used to inactivate *stlB* and *chlA* have not affected the partner gene. We, therefore, conclude that StlB is required to produce all the chlorinated compounds produced in development.

Taken together these results show that developing *D. discoideum* cells produce an abundant set of chlorinated compounds, predominantly in the mature fruiting body. They are likely synthesized by StlB and ChlA, the same enzymes as produce DIF-1 earlier in development. However, since none of these late chlorinated compounds have been chemically identified, the changes to the DIF-1 biosynthetic pathway required to produce them remain unknown.

### (b) Chemical identification of a late chlorinated compound, CDF-1

**Figure 2.**
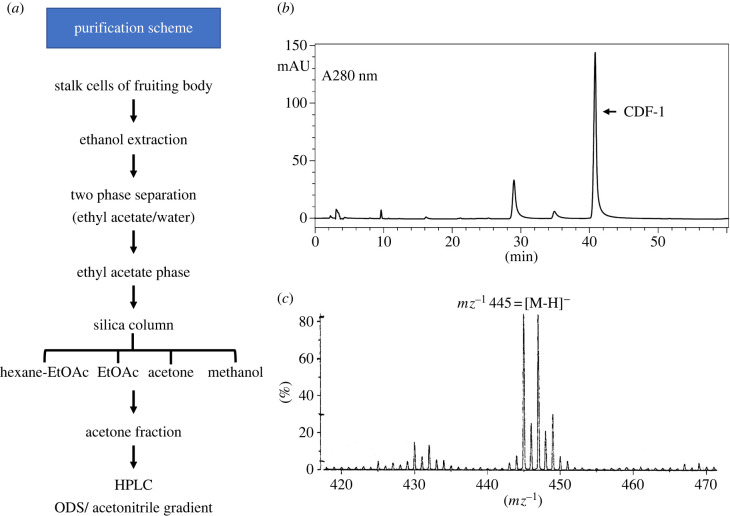
Purification and characterization of CDF-1. (*a*) Purification scheme. (*b*) HPLC profile from the last step of the purification. The acetone fraction from SiO_2_ column chromatography was analysed by reverse-phase HPLC (TSK gel ODS-120T) eluting at 1 ml min^−1^ with a gradient of 50–80% acetonitrile in 60 min (*c*) Mass-spectroscopic analysis of CDF-1 (FAB-MS in negative ion mode).

In order to identify one or more of the late chlorinated compounds we optimized their production and devised a purification scheme. We found by ^36^Cl^−^ labelling that strain V12M2 produced about 5 times more of these compounds than the standard axenic strain, Ax2 (electronic supplementary material, figure S1), and that they could be efficiently extracted from stalk cells using ethanol. The extracted chlorinated compounds were purified as outlined in figure 2*a* and Materials and Methods, using TLC to monitor the fractions.

In one preparation 490 g wet weight of mature stalks yielded 5.56 g of ethanolic extract (dry weight), which, after partitioning between ethyl acetate and water, yielded 1.02 g in the organic phase. Repeated SiO_2_ column chromatography yielded 1.2 mg of a purified chlorinated compound, which we call CDF-1 (chlorinated dibenzofuran-1). A second purification yielded 0.9 mg of CDF-1 from 171.5 g (wet weight) of stalk cells.

Mass spectroscopy of CDF-1 (FAB-MS using a JEOL JMS-700) in negative ion mode showed four molecular ion peaks, *m/z* 445 447 449 and 451, in a ratio of 27:27:9:1, suggesting that CDF-1 contains three chlorine atoms (figure 2*c*). HRFAB-MS measurement with negative ion mode provided *m*/*z* 445.0003, consistent with a molecular formula for CDF-1 of C_19_H_17_Cl_3_O_6_ (calculated for C_19_H_16_Cl_3_O_6_ [M-H]^−^ 445.00125). The full chemical structure was elucidated by ^1^H-NMR, ^13^C-NMR, HMBC, HMQC and COSY spectra (JEOL JNM-ECA 500). These data are summarized in electronic supplementary material, figure S5–S7.

**Figure 3.**
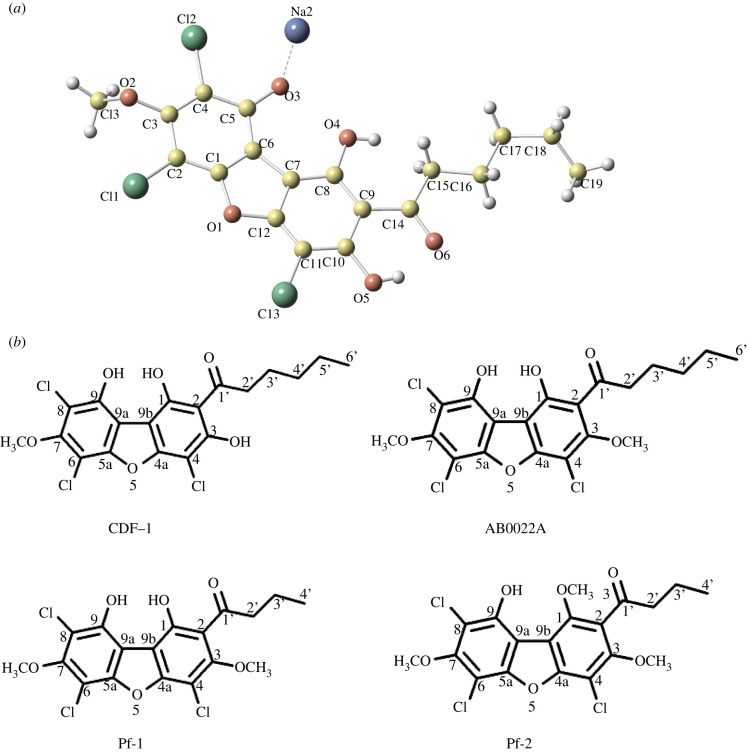
Structure of CDF-1 and related natural products. (*a*) Space-filling model of CDF-1 as the sodium salt. Ethyl acetate (the solvent) is omitted for clarity. (*b*) CDF-1 and dibenzofurans from other slime mould species.

CDF-1 formed yellow, needle-like crystals by vapour diffusion from an ethyl acetate solution at 22°C with hexane as the reservoir solution. This allowed its molecular structure to be unambiguously determined by X-ray crystallography (CCDC-2 099 186; electronic supplementary material, table S2) as a chlorinated dibenzofuran (figure 3*a*). Similar chlorinated dibenzofurans are produced by other cellular slime moulds (figure 3*b*): Pf-1 and Pf-2 from *P. filamentosum*, and AB0022A from *D. purpureum*, which only differs by a methyl group from CDF-1 [23,24].

### (c) Synthesis of CDF-1 from the polyketide THPH

**Figure 4.**
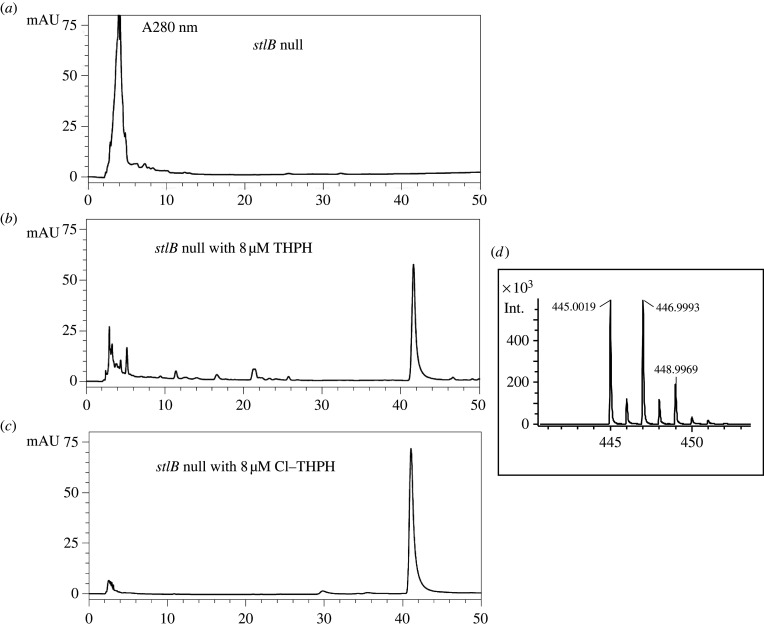
Cells can utilize the polyketide THPH or its chlorinated derivative Cl-THPH to make CDF-1. Axenically grown *stlB* null cells were collected, washed and spread on 10% DIFlab agar containing 100 nM DIF-1, with or without THPH or Cl-THPH. Cells were allowed to make fruiting bodies on agar for 3 days, then extracted with ethanol, partitioned between ethyl acetate and water and the extracted molecules purified through SiO_2_ column chromatography and HPLC to detect CDF-1. (*a*) HPLC profile of *stlB* null stain, (*b*) HPLC profile of *stlB* null strain incubated with 8 µM THPH, (*c*) HPLC profile of *stlB* null strain incubated with 8 µM Cl-THPH, (*d*) ESI-MS with negative ion mode analysis of CDF-1 peak isolated in (*c*).

Our data indicate that the polyketide precursor of CDF-1 is made by StlB. Since the only polyketide known to be produced by this enzyme is THPH, we asked whether THPH is also a precursor of CDF-1. *StlB-* cells were developed with a supply of THPH and ^36^Cl-labelling used to detect chlorinated products. To ensure that the cells developed well, 100 nM DIF-1 was also included in the agarose. The results clearly show that *stlB-* cells can use the supplied THPH to make a series of chlorinated compounds that they cannot make without it (figure 1*c*). We used HPLC and ESI-MS mass-spectroscopy to confirm that one of these is CDF-1 (figure 4*a,b*). In further experiments we fed mutant cells with the mono-chlorinated derivative of THPH (Cl-THPH) and found that it also serves as a precursor for CDF-1 (figure 4*c,d*).

These experiments show that the same polyketides serve as precursors for both DIF-1 and CDF-1. This raises the twin questions of how the biosynthetic pathway using THPH switches during development to produce first DIF-1 and then CDF-1; and how is the cellular context reprogrammed to direct synthesis to the stalk?

### (d) Cellular reprogramming for CDF-1 production

DIF-1 is made by prespore cells during multicellular development, prior to fruiting body formation [29], but in contrast, CDF-1 accumulates in the stalk cells of the mature fruiting body, not in its spores [25]. The mRNAs for StlB and ChlA first accumulate when DIF-1 is made, then decline, before a strong increase late in development [20]. These genes are adjacent in the genome and share a common promoter region. We, therefore, used an EGFP construct driven by promoter sequences adjacent to each gene to ask where they are expressed during development.

**Figure 5.**
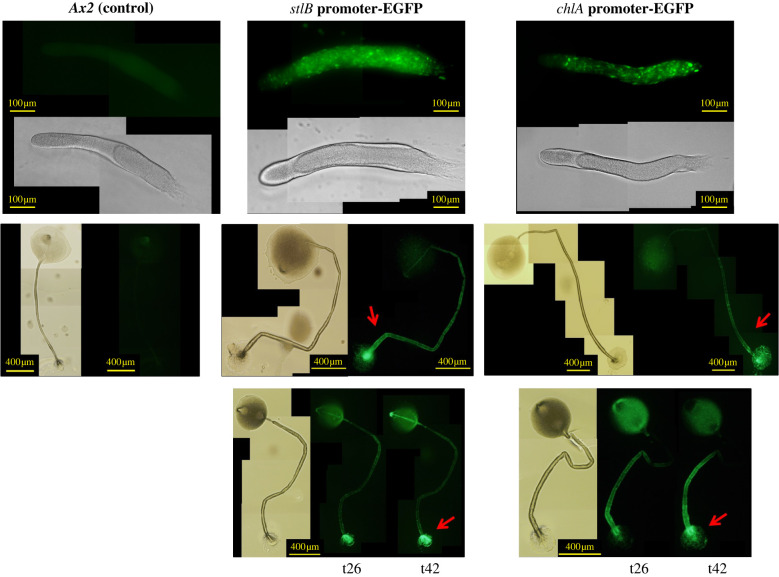
Spatial expression of the *stlB* and *chlA* genes. Both genes are expressed in the prespore region of slugs, but in the stalk and basal disc of fruiting bodies. Ax2, the parental strain, was used as negative control. Promoters of the two genes were used to drive EGFP expression. Red arrow indicates the basal disc.

Figure 5 shows that in migrating slugs, where DIF is made, both *stlB* and *chlA* are expressed in the prespore region, consistent with earlier work. By contrast, later in development expression switches to the stalk and basal disc regions of mature fruiting bodies.

**Figure 6.**
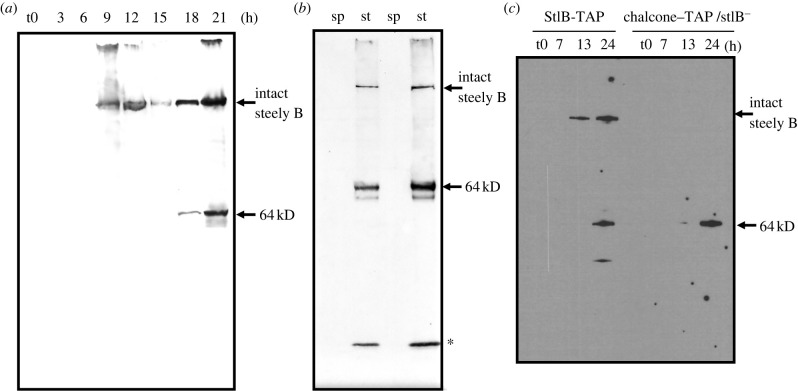
Stalk cells release the chalcone synthase domain of Steely-B. Western blots probed for the TAP tag of the StlB-TAP protein. (*a*) Developmental expression of the TAP-tagged StlB protein (about 352 kDa). StlB expression peaked at 9–12 h of development, as aggregates consolidated and developed tips, then declined, before a second peak of strong expression as fruiting bodies formed. In this second phase a smaller band (about 64 kDa) is also detected, corresponding to the released C-terminal chalcone synthase domain. (*b*) StlB-TAP expression is limited to the stalk of mature fruiting bodies, where the shorter, chalcone synthase domain dominates. Stalk and spores were separated from mature fruiting bodies by washing through a mesh. st: stalk; sp: spore. (*c*) Expression of the isolated, TAP-tagged chalcone synthase domain (indicated as ‘chalcone-TAP’) of StlB in a *stlB* null strain. The expressed protein is of the expected size and expression occurs late in development. Asterisk indicates unknown degradation product.

To examine StlB protein expression, we knocked a TAP tag into the C-terminus of the protein at the endogenous locus, and confirmed that development and labelling with ^36^Cl^−^ is normal in the resulting strain (electronic supplementary material, figure S3). Western blots of the tagged strain show the expected developmental regulation of the StlB protein, with a peak in mid-development, followed by a second one later, as stalk cells form (figure 6*a*). Separating stalk and spores from mature fruiting bodies showed the protein is specific to stalk cells, not spores (figure 6*b*).

Unexpectedly, we found that the protein is processed late in development to release a 64 kDa fragment, still tagged with TAP and therefore corresponding to the C-terminal chalcone synthase domain (42.7 kDa) which with the tag (21 kDa) is approximately 64 kDa [30]. This fragment is the predominant form in stalk cells, raising the possibility that it produces the polyketide precursors of the late chlorinated compounds. The short form of StlB is unlikely to be due to alternate splicing because there is no intron in the 3′ region of the *stlB* gene. In addition, RT-PCR shows that the type I and type III PKS domains of StlB have the same expression profiles.

**Figure 7.**
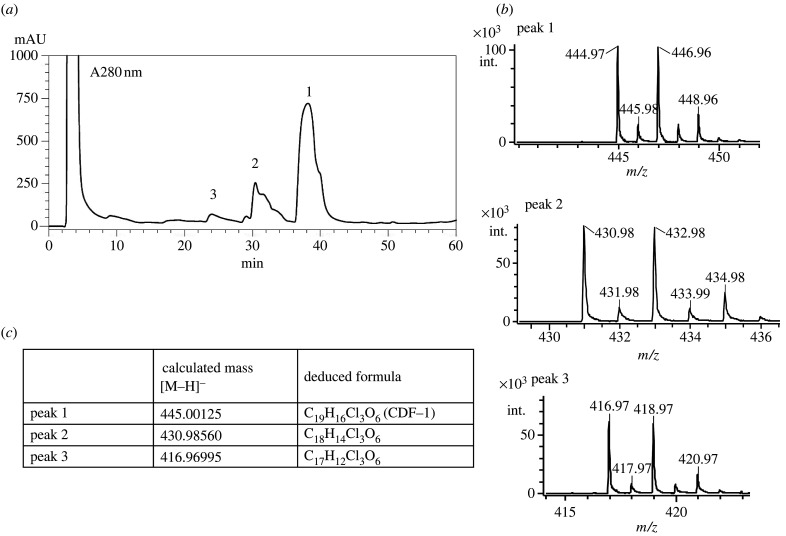
The isolated chalcone synthase domain of Steely-B supports synthesis of CDF-1. Extracts from fruiting bodies of cells expressing the chalcone synthase domain of StlB were analysed for production of CDF-1. (*a*) HPLC profile of the partially purified extract, (*b*) Negative ion mode ESI-MS analysis of each peak from (*a*). (*c*) Calculated mass and deduced formula of CDF-1 and other chlorinated compounds produced by *stlB* null cells expressing the isolated chalcone synthase domain. Peak 1 corresponds to CDF-1, while peaks 2 and 3 are consistent with derivatives lacking one or two methylene moieties. The TAP-tagged chalcone synthase domain was expressed in a *stlB* null mutant under the control of the stalk-specific ST promoter. Cells were grown with *K. aerogenes* on 0.5xSM agar in 25 × 25 cm plastic plates, with 120 plates per experiment. Neither DIF, nor DIF precursors, were added in the agar. Mature fruiting bodies were collected, extracted with ethanol and partitioned between ethyl acetate and water. The ethyl acetate extract was separated by preparative SiO_2_ column chromatography to yield the partially purified extract, which was analysed by HPLC and mass-spectroscopy.

To test whether the chalcone synthase domain alone is sufficient to produce CDF-1, we expressed this domain in *stlB* null cells, using a promoter that ensured its production in stalk cells. The fruiting body morphology of this mutant is the same as that of the *stlB* knockout mutant, having the typical ‘DIF-less’ phenotype of defective slugs and fruiting bodies lacking a basal disc, with spores at their base. Western blotting showed that the TAP-tagged chalcone synthase domain is only expressed late in development and is the same size as the fragment released from the intact protein (figure 6*c*). Mass spectroscopy shows that these cells produce CDF-1 and related compounds, two of which had empirical formulae suggesting they have one or two fewer methylene moieties than CDF-1 (figure 7). These could be derivatives with shorter alkyl tails.

These experiments show that there is developmental reprogramming of polyketide synthesis in which StlB and ChlA are expressed in stalk cells late in development, where StlB is predominantly cleaved to release the chalcone synthase domain. This domain alone can support the production of CDF-1: presumably this is via incorporation of an hexanoyl starter unit, leading to the formation of THPH after three rounds of malonyl-CoA utilization.

### (e) Anti-bacterial activity of CDF-1

The biological function of CDF-1 remains a mystery. It is unlikely to participate in the developmental process producing fruiting bodies, which is essentially complete before it is made in any substantial amount. One potential ecological role would be to protect the dormant spores from bacterial or other infection. We therefore tested whether it has antibacterial activity. Table 2 shows that CDF-1 has strong activity against Gram-negative *E. coli* and Gram-positive *B. subtilis* for which it has a potency comparable to ampicillin. Compound AB0022A from *D. purpureum*, which is chemically very similar to CDF-1, had no activity against *E. coli*, showing the importance of the hydroxyl group at the 3 position [24].

**Table 2.** Antibacterial activity of CDF-1. Ampicillin was used as a positive control. CDF-1 showed similar antibacterial activity with that of Ampicilin.

tested bacteria	antibiotic	minimum inhibitory concentration (μg ml^−1^)
*B. subtilis*	ampicillin	0.47
	CDF-1	0.19
*K. aerogenes*	ampicillin	>120
	CDF-1	>100
*E. coli B/r*	ampicillin	3.75
	CDF-1	6.25

## 4. Discussion

Here we show that the abundant chlorinated compounds made in the stalk of the *Dictyostelium* fruiting body [25] all depend on the StlB polyketide synthase and ChlA chlorinating enzyme for their synthesis, just like DIF earlier in development [14,20]. Our chemical identification of one of these stalk compounds, shows that it is a chlorinated dibenzofuran and so not an obvious metabolite of DIF, which form by dechlorination and P450-dependent oxidation [31,32]. However, the same polyketide as produces DIF-1 is also used to produce CDF-1, implying that there is a change in the downstream processing pathway.

The developmental switch from DIF-1 to CDF-1 production is accompanied by a cellular re-programming in which expression of the StlB PKS and ChlA chlorinating enzyme is shifted from prespore cells to stalk cells, and StlB is cleaved to release the C-terminal chalcone synthase domain as the major product. We show that this cleaved domain can still support CDF-1 synthesis, presumably by employing hexanoyl-CoA supplied by one or more of the other FAS/PKS proteins.

The full biosynthetic pathway of CDF-1 remains to be discovered. Since half of the CDF-1 polyketide skeleton is the same as THPH, one possible pathway is the oxidative phenolic coupling of THPH to a phenolic compound such as phloroglucinol. Another possibility is that two molecules of THPH make the dibenzofuran backbone. In this case, the acyl chain of one THPH needs to be cleaved off. The C-C bond between an aryl moiety and a ketone in aryl ketones can be hydrolyzed by Friedel-Crafts hydrolases. Four different Friedel-Crafts hydrolases are known in bacteria and fungi, but we could not detect any homologues in the *Dictyostelium* genome [33].

Previous work shows that there are three chlorinated compounds of high abundance produced by mature fruiting bodies and further lesser ones [25], only one of which have we identified here. The unidentified compounds also require StlB and ChlA for their synthesis and are therefore probably closely related to CDF-1. They may also be synthesized by the isolated chalcone synthase domain, but using different acyl-CoA precursors from CDF-1: release of the domain may relax its specificity by breaking the channeling of precursors from the type-I PKS region of StlB. Such relaxed specificity is seen with the bacterially expressed chalcone-synthase domain of StlB, which can accept isovaleryl-CoA, hexanoly-CoA and heptanoyl-CoA [14].

As denizens of the soil, *Dictyostelium* amoebae live in a complex environment with a wide range of competitors, prey and predators. The richness of polyketide synthase genes in their genomes and the specialization of these genes in related species suggests that polyketides are widely used by amoebae to mediate ecological interactions [6]. Although *Dictyostelium* amoebae are predators on bacteria during their growth phase, it appears likely that in their development they may fall prey to bacterial infection. Accordingly, they have evolved a form of innate immunity that protects the migrating slug stage from bacterial infection [34], and the antibiotic activity of CDF-1 that we have identified may give protection to the mature fruiting body.

## 5. Conclusion

Small molecules, including polyketides, mediate many interactions between organisms in the soil eco-system. *Dictyostelium* amoebae are richly endowed with polyketide synthase genes, including the unique Steely fusions. We show that their polyketide diversity is increased by a dual function of SteelyB. During *D. discoideum* development it first makes a signal called DIF and later an abundant chlorinated dibenzofuran called CDF-1. This reprograming is due both to altered expression and processing of the StlB protein. CDF-1 is a potent anti-bacterial and we speculate it may protect the fruiting body from infection.

## Data Availability

The datasets supporting this article have been uploaded as part of the electronic supplementary material [35].
